# Prevalence of chronic periodontitis in an obese population: a preliminary study

**DOI:** 10.1186/s12903-015-0098-3

**Published:** 2015-09-29

**Authors:** Shahrukh Khan, Roslan Saub, Rathna Devi Vaithilingam, Syarida Hasnur Safii, Shireene Rathna Vethakkan, Nor Adinar Baharuddin

**Affiliations:** Department of Restorative Dentistry, Faculty of Dentistry, University of Malaya, Lembah Pantai, 50603 Kuala Lumpur, Malaysia; Department of Community Oral Health & Clinical Prevention, Faculty of Dentistry, University of Malaya, Lembah Pantai, 50603 Kuala Lumpur, Malaysia; Department of Medicine, Faculty of Medicine, University of Malaya, 50603 Kuala Lumpur, Malaysia

**Keywords:** *Chronic periodontitis*, *Obesity*, *BMI*

## Abstract

**Background:**

Chronic periodontitis (CP) is a global public health issue. Studies have suggested CP could be linked to obesity due to their similar pathophysiological pathway. The aim of this study is to determine the prevalence of CP and to assess the predictors for CP among the obese Malaysian population.

**Methods:**

This is a cross-sectional study on obese participants. Obesity is defined as an individual who has Body Mass Index (BMI) ≥27.5 kg/m^2^. A convenience sampling method was used. A total of 165 paricipants were recruited. This study involved answering questionnaires, obtaining biometric and clinical measurements of Visible plaque index (VPI), Gingival bleeding index (GBI), Probing pocket depth (PPD) and Clinical attachment loss (CAL). Data analysis was carried out using SPSS statistical software (SPSS Inc., version 20, US).

**Results:**

A total of 165 participants; 67 (40.6 %) males and 98 (59.4 %) females participated in the study. Mean age of the participants was 43.9 (±8.9). The prevalence of CP among the obese population was found to be 73.9 %. Out of this, 43 and 55 % were categorised as moderate and severe CP respectively. Around 64 % of participants had sites with CAL ≥4 mm and participants with sites with PPD ≥4 mm were reported to be 25 %. Around 83 % of the participants had sites with GBI ≥30 and 92 % of participants had sites with VPI ≥20 %. GBI and VPI were found to have significantly higher odds for CP.

**Conclusion:**

Prevalence of CP was high among obese Malaysians. GBI and VPI were potential predictors for CP in this obese population.

## Background

Chronic periodontitis (CP) is a major oral health problem and it is considered as one of the reasons for tooth loss in developing and developed nations. Worldwide, the prevalence of CP in the general adult population is reported to be 30–35 %, with approximately 10–15 % diagnosed with severe CP [1]. In Malaysia, the prevalence of the CP and severe CP was reported as 48.5 and 18.2 % respectively [2]. Studies have suggested that CP may have a negative impact on the quality of life (QoL) of the affected adults and this could include difficulty in chewing, speaking, or social interactions [3]. Various risk factors have been identified associated with CP such as diabetes mellitus, cardiovascular diseases, smoking and obesity [4].

Obesity is a major public health concern in both developing and developed countries. The prevalence of obesity among adults was estimated at 24 % worldwide [5], 10–30 % in South East Asia [6] and 27.2 % in Malaysia [7]. Obesity is a potential risk factor for major complex diseases such as diabetes, cardiovascular diseases, metabolic syndromes as well as CP [8]. The relationship between obesity and CP was first reported in experimental animals [9]. Subsequent human studies have confirmed that obesity increases risk for CP [10, 11]. A 5 year longitudinal study conducted in Japanese workers demonstrated a positive relationship between BMI and periodontal disease [12]. A systematic review based on 26 cross sectional studies, 6 case control studies and 1 cohort study concluded that there was a positive association between obesity and periodontitis [13].

To date, there is no published study on the prevalence of CP among the obese Malaysian population and only limited published information in the South East Asia region. The aim of this study was to determine the prevalence of CP and its predictors in the obese Malaysian population.

### Methods

#### Study design and population

This is a cross sectional study on the obese participants. Obesity is defined as an individual who has body mass index (BMI) ≥27.5 kg/m^2^ based on the Asian classification of BMI [14]. A convenience sampling method was used for the participant’s recruitment. The inclusion criteria consisted of the obese participants, aged 30 years and above and have at least 12 teeth present. Participants who had received periodontal treatment within the last 4 months and who had been on the antibiotics within the past 4 months were excluded, also those participants who required prophylactic antibiotic coverage or those on systemic or topical steroidal anti-inflammatory drugs for the past 4 months, pregnant or lactating mothers and those with learning disability were excluded.

Participants who fulfilled the inclusion and exclusion criteria were invited to participate. Participants were recruited for a period of 9 months from February 2013 until October 2013. Participants were recruited through three different routes i.e. Obesity clinics (University Malaya Medical Center), Primary Care Unit (Faculty of Dentistry, University of Malaya) and from the Malaysian Periodontal Database and Bio bank System [15]. The nature of the study was explained and a written informed consent was obtained from participants who agreed to take part in the study. Ethical approval was obtained from the Medical Ethical Committee Faculty of Dentistry (DFOP 1213/0079 (L)) and the University Malaya Medical Centre (MEC 96223). This research was carried out in accordance with the World Medical Association, Declaration of Helsinki guidelines. All participants (i) answered the questionnaire and (ii) underwent clinical assessments (biometric and clinical measurements).

#### Questionnaire

The questionnaire covered the socio-demographic of participants (age, gender, ethnicity and level of education), habits of smoking and alcohol intake, tooth brushing frequency and last dental visit. The participants were divided into three subgroups based on age i.e. (i) 30–39 years, (ii) 40–49 years and (iii) 50 and above. Ethnic groups consisted of Chinese, Malays and Indians. Education level was assessed as primary, secondary or tertiary level of education. Smoking habit was categorized into two sub-categories: (i) current smokers and (ii) former/never smokers. Alcohol intake was classified as: (i) yes (alcohol users) and (ii) no (non-alcohol users). The participants were classified into 3 categories based on their last dental visits as those (i) who last visited a dentist <2 years, (ii) who last visited a dentist ≥2 years and (iii) who never visited a dentist. Tooth brushing frequency was classified as participants who brush their teeth, (i) ≥2times per day and (ii) once daily.

#### Clinical assessment

### Biometric measurement

The biometric assessment was carried out using the body mass index (BMI) as a measure of obesity. The height (in meter) and body weight (in kg) were measured and used to calculate the BMI using the formula below:

BMI = Body weight (kg)/Body height^2^ (m^2^)

Obesity was subcategorised based on the BMI as Obese I i.e. those with 27.5–34.9 kg/m^2^, Obese II i.e. those with 35–39.9 kg/m^2^ and Obese III i.e. those with BMI ≥ 40 kg/m^2^.

### Clinical measurements

A full mouth periodontal measurement (FMPM) included Visible Plaque Index (VPI) [16], Gingival Bleeding Index (GBI) [16], Probing Pocket Depth (PPD), Recession (R) and Clinical attachment loss (CAL) assessment. A William’s periodontal probe (Nordent, US) with calibrated markings was used and measurements were recorded in millimetres (mm). The FMPM were carried out on all teeth present except third molars. Six sites per tooth were examined which included mesio-buccal, mid-buccal and disto-buccal and also the mesio-palatal/lingual, mid-palatal/lingual and disto-palatal/lingual surfaces. VPI and GBI were recorded using dichotomous score.

CP was defined based on the case definition as shown in Table 1 [17]. Three examiners carried out the FMPM (SK, ZA and SB). Standardisation and calibration were carried out. Inter- and intra-examiner reproducibility was conducted. Kappa scores were estimated as 0.76 and 0.82 for inter-examiner and intra-examiner reliability respectively.

**Table 1:** Case Definition Proposed for Population-Based Surveillances of Chronic Periodontitis by Eke et al. [17]

Case	Definition
No chronic periodontitis	No evidence of mild, moderate or severe periodontitis
Mild chronic periodontitis	≥2 interproximal sites with CAL ≥3 mm and ≥2 interproximal sites with PPD ≥4 mm (not on same tooth) or one site with PPD ≥5 mm
Moderate chronic periodontitis	≥2 interproximal sites with CAL ≥4 mm and ≥2 interproximal sites with PPD ≥5 mm (not on same tooth)
Severe chronic periodontitis	≥2 interproximal sites with CAL ≥6 mm (not on same tooth) and ≥1 interproximal site with PPD ≥5 mm

### Data analysis

Statistical analysis was carried out using statistical packages for social sciences software (SPSS Inc., version 20, US). The characteristics of participants were assessed using frequency distribution for categorical variables and mean (standard deviation, ±) for continuous variables. The frequency distribution was used to estimate prevalence of CP in relation to socio-demographics, habits, last dental visit, tooth brushing frequency and BMI indicator of obesity. The cross tabulation was carried out for VPI, GBI, PPD ≥4 mm and CAL ≥4 mm in relation to CP. Mean (standard deviation “±”) were calculated for mean PPD and mean CAL. Multivariate binary logistic regression analysis was used to identify the predictors for CP in obese population, which includes social demographic factors (gender, level of education and ethnicity), smoking and alcohol habits, last dental visit, tooth brushing frequency, level of BMI and periodontal parameters (GBI and VPI).

## Results

A total of 165 obese participants with mean age 43.9 (±8.9) participated in the study. The participants’ age ranged from 30 to 66 years. Table 2 summarizes the socio-demographic characteristics, habits and BMI of the participants and its CP prevalence. On the basis of the characteristics of the participants, around 70 % of the participants were in an age range of 30–49 years. Females were predominant part of the sample (59.4 %). Around 77 % of the participants were Malays. Almost 93 % of the participants had at least secondary education. Smoking and alcohol intake were found uncommon characteristics of the sample population. About 6 out of 10 participants last visited a dentist more than 2 years ago. Most of the participants (84.8 %) brushed their teeth twice daily. Around 70 % of the participants were in Obese I category.

**Table 2:** Socio-demographic characteristic s, habits and BMI distribution among obese and their CP prevalence

Social-demographic characteristics	*N* = *165 n* (%)	*Prevalence of CP N = 122 n (%)*
Age		
• 30–39	65 (39.4)	45 (36.9)
• 40–49	51 (30.9)	37 (30.3)
• 50 above	49 (29.7)	40 (32.8)
Gender		
• Male	67 (40.6)	47 (30.5)
• Female	98 (59.4)	75 (61.5)
Ethnicity		
• Malay	127 (77)	91 (74.5)
• Non Malays	38 (23)	31 (25.5)
Level of Education		
• Primary	12 (7.3)	11 (9.0)
• Secondary and Tertiary	153 (92.7)	111 (91.0)
Smoking		
• Current	26 (15.8)	21 (17.2)
• Former/Never	139 (84.2)	101 (82.7)
Alcohol		
• Yes	13 (7.9)	11 (9.0)
• No	152 (92.1)	111 (91.0)
Last Dental Visits		
• <2 years	42 (25.5)	30 (24.6)
• ≥2 year	96 (58.2)	76 (62.3)
• Never	27 (16.4)	16 (13.1)
Tooth Brushing frequency		
• ≥2times/day	140 (84.8)	101 (82.8)
• 1 time/day	25 (15.2)	21 (17.2)
BMI^a^		
• Obese I	116 (70.3)	81 (66.4)
• Obese II	32 (19.4)	26 (21.3)
• Obese III	17 (10.3)	15 (12.3)
CP		
• Yes	122 (73.9)	
• No	43 (26.1)	
CP Severity		
• Moderate CP	53 (43.4)	
• Severe CP	67 (54.9)	

^a^Obese I = 27.5–34.9, Obese II = 34.9–39.9, Obese III = 40 and above

*CP* chronic periodontitis

In this study, the prevalence of CP among the obese participants was reported to be 73.9 %. Out of this, 43.4 and 55 % were moderate and severe CP respectively. Of those with CP, almost 40 % was those in the young age group (30–39 years) and almost two third were females. Around 23 % obese participants with CP were non-Malays and around 91 % obese participants with at least secondary education had CP. Obese smokers and alcohol users had a CP prevalence of 17.2 and 9 % respectively. Around 62 % of the obese participants with CP had last dental visit more than 2 years ago. Almost 83 % of obese participants with CP brushed their teeth twice daily.

Table 3 shows the distribution of periodontal parameters in relation to CP among obese participants. Around 83 % participants had sites with GBI ≥30 %. Eighty percent of CP patients had sites with VPI ≥ 20 %. The distribution of sites with PPD ≥4 mm and CAL ≥4 mm were estimated as 25.4 and 63.9 % respectively. The mean scores of PPD and CAL were 3.1 (±0.8) and 3.6 (±0.6).

**Table 3:** Periodontal parameters in relation to CP

Periodontal parameters	CP *N* = 122 Number of participants *n* (%)
GBI	
<30 % sites	21 (17.2)
≥30 % sites	101 (82.8)
VPI	
≤20 % sites	10 (8.2)
≥20 % sites	112 (91.8)
PPD ≥4 mm	31 (25.4)
CAL ≥4 mm	78 (63.9)
	Mean (±)
PPD	3.1 (±0.8)
CAL	3.6 (±0.6)

*GBI* Gingival Bleeding Index, *VPI* Visible plaque index, *PPD* Probing pocket depth, *CAL* Clinical attachment loss, SD “±”, *CP* chronic periodontitis

Table 4 shows the logistic regression of the socio-demographic characteristics, habits and periodontal parameters in relation to the prevalence of CP in the obese population. No significant differences were noted for socio-demographics, habits and level of BMI. Participants who last visited a dentist ≥2 years were found to have significantly higher risk for having CP. The sites with GBI ≥30 % and VPI ≥20 % were found to have significantly strong risk of having CP.

**Table 4:** Logistic regression showing socio-demographic characters, habits and periodontal parameters in relation to CP

Predictors	CP OR (95 % CI)
Gender	
• Male	1 (Reference)
• Female	0.7 (0.3–1.4)
Level of education	
• Primary	1 (Reference)
• Secondary and Tertiary	0.5 (0.2–1.2)
Ethnicity	
• Non Malays	1 (Reference)
• Malays	0.5 (0.2–1.2)
Smoking	
• Yes	1 (Reference)
• No	0.6 (0.3–1.3)
Alcohol	
• Yes	1 (Reference)
• No	0.5 (0.2–1.7)
Last Dental Visits	
• <2 years	1 (Reference)
• ≥2 year	2.6 (1.4–6.5)*
• Never	1.7 (0.6–4.7)
Tooth Brushing frequency	
• ≥2times/day	1 (Reference)
• 1 time/day	0.9 (0.3–2.4)
BMI	
• Obese I	1 (Reference)
• Obese II	1.8 (0.7–4.9)
• Obese III	3.2 (0.7–8.9)
GBI sites	
• ≤30 %	1 (Reference)
• ≥30 %	5.0 (2.3–10.7)*
VPI sites	
• ≤20 %	1 (Reference)
• ≥20 %	4.3 (1.7–10.9)*

*p < *0.001*

*GBI* Gingival Bleeding Index, *VPI* Visible plaque index, *PPD* Probing pocket depth, *CAL* Clinical attachment loss, SD “±”, *CP* chronic periodontitis, *BMI* Body mass index

## Discussion

The present study found that the prevalence of CP among the obese Malaysian population was almost 74 %. In Malaysia, there has been a rising trend in the prevalence of CP over the years among the general population. The NOHSA 2010 estimated the prevalence of CP to be 48.5 %, which is almost two folds of that which was reported by NOHSA 2000 (25.2 %) [2]. Even the globally reported prevalence of CP is 30–35 % among the general adult population [1].

Studies in the Jordanian and the USA populations, reported that the prevalence of CP among the obese was 51.9 and 35 % respectively [10, 11]. Although their prevalence of CP was lower than the Malaysian obese population, nonetheless, they concurred that obese participants have a higher CP prevalence compared to their general populations. In this study, out of the 73.9 % obese participants with CP, 54.9 % participants were categorised as severe stage of CP. This is alarmingly high compared to only 18.2 % reported in the general Malaysian population [2]. The worldwide prevalence of severe CP in the general adult population was approximately between 10–15 % [1].

Since the population of the present study are obese, it could be speculated that obesity could have contributed to the increased burden of inflammation through increased expression of pro-inflammatory cytokines [18]. Obesity is associated with production of pro-inflammatory cytokines that may play a role in the already existing burden of inflammation associated with CP [19].

This study found around 36.8 % of participants who had CP came from the 30–39 years old age group. This finding differed from previous study on general populations whereby higher pevalence of CP was associated with older age groups [20]. It could be speculated that the high prevalence of CP among this age group could be due to the fact that this group belongs to the working age group. They could be preoccupied with their daily routine, thus, they do not have time to visit the dental clinic regularly. The busy lifestyle among the younger adults also could have indirectly induced stress which may have contributed to the overall burden of inflammation [21]. In the present study, the prevalence of CP was much higher in females as compared to male participants. Under obese conditions, females and males are both equal in terms of obesity-induced burden of inflammation. However, females experience changes in hormonal levels of estrogen and progesterone during pre-menstrual and menstrual phases [22]. A study by Machtei et al. (2004) among Israeli women found that such an increase in hormonal levels have a negative impact on the periodontal status [23]. This could be explained based on the fact that hormones modulate changes through alterations in the host immune response and cellular functions in gingival tissues [22].

In the present study, around 17 % of the obese participants who smoke had CP. This finding disagrees with previous study conducted in the Swedish population, reporting a high prevalence of CP with smoking habit [24]. Smoking has the potential to affect the host response at the cellular, vascular and tissue repair level including alteration in neutrophil function, antibody production, fibroblast activities, vascular factors and inflammatory mediator production, thus, supresses the host healing ability and contributes to disease accumulation and progression [4]. However, this finding was not reflected in the obese participants in the current study probably due to the small sample size of the study population. This makes it difficult to establish a pattern between CP with smoking habits among the obese participants.

This study reported percentages of population with CAL ≥4 mm as 64 %. No data was reported in previous obese population studies to allow further comparison. Meanwhile, among various populations, percentages of CAL ≥4 mm vary between 48 to 84 % [25, 26]. This finding is expected because CAL is an indicator of past disease experience and also an estimate of accumulative periodontal tissue destruction [27]. The extent of periodontal tissue destruction would therefore depend on how long the investigated population has been exposed to the disease.

In this study, almost 25 % of the obese individuals had PPD ≥4 mm. Studies among different populations have reported that about 20–28 % of their participants have PPD ≥4 mm [26, 28, 29]. The finding of the current study suggests that regardless of the state of obesity, exposure to periodontal disease may depend upon the susceptibility of an individual’s genetic makeup [30]. Variation in periodontal measures can be a result of genetic factors. The USA twin based survey conducted among 117 pairs of adult twins showed 50 % susceptibility of CP is associated with genetics [30]. This suggests that the heritable nature of CP is biologically possible and reflects genetically determined variations in host immune responses [30].

Unlike CAL, PPD is the periodontal measurement of active disease and provides information on current disease status [27]. However, the measurement of PPD is not as reliable as the CAL. It depends on various factors such as inflammatory condition of the marginal gingiva.

Almost 83 % participants in this study had sites with GBI ≥30 %. There are no comparative studies reporting bleeding scores in other obese populations. Previous studies conducted in Danish and Thai general adult populations reported 90–95 % individuals having BOP [28, 31]. This shows that regardless of obesity, the participants in the current study had gingival inflammation which is an indicator of the presence of CP instability and the existence of inflammation. BOP is a sign of gingival inflammation. Persistent BOP is a positive indicator for CP progression by 30 % [32]. On the other hand, absence of BOP was a negative predictor for CP by 96 % [33].

In the present study, 91.8 % participants had sites with VPI ≥20 %. Dental plaque is the etiological factor for CP [34]. Since 74 % participants in the current study had CP, it was expected that participants in the current study would have high VPI scores. A Brazilian population based study among CP patients reported almost 86.5 % of participants with plaque accumulation [35]. This concurs with the fact that plaque accumulation is a pre-requisite for gingival inflammation and CP [34].

The current results showed that those who had their last dental visit more than 2 years ago was found to be significantly associated with CP (*p* < 0.001). The current finding disagrees with the US study on dentate diabetic adults that showed no significant influence of dental visit pattern on periodontal disease status of the participants [36]. The reason for irregular dental visits in the current study could be due to lack of awareness on the importance of regular dental visits. Also, dental anxiety and fear could have contributed to irregular dental visit [2]. Furthermore, an increase in cost of dental treatment with no provision of dental care insurance could have influenced the association further [37].

The current study also showed no significant association between tooth brushing frequency and CP. This finding is in disagreement with previous studies which reported significantly higher odds ratios for the association between tooth brushing frequency and CP [38]. Tooth brushing is an important measure for plaque control [39]. However, efficient tooth brushing technique has been shown to be the primary factor in obtaining optimum plaque control, rather than the frequency of tooth brushing [40]. In addition, the participants in the current study suffered from moderate to severe CP. Therefore, tooth brushing alone may not be sufficient to prevent further deterioration of periodontal health. Provision of non-surgical periodontal treatment would benefit further in controlling the CP progression [41].

The current study showed that the increase in obesity level had no significant association with CP in this obese sample of Malaysians. This finding was in disagreement with studies conducted in the Japanese, Jordanian and the USA populations, where increased BMI was found associated with periodontal disease. The findings of Al-Zahrani et al. (2003), reported an increase in BMI (above 30 kg/m^2^) was associated with increased prevalence of CP [10]. Khader et al. (2009) also showed significant associations between BMI and CP [11]. The biological plausability for the link between obesity and CP is yet to be established. However, obesity is a state of chronic inflammation, and increase in BMI levels has been found to be associated with increased adipokine levels. These inflammatory adipokines like TNFα and IL-6 contribute to the pre-exiting inflammatory state associated with CP and breakdown of periodontal tissue support [18].

Participants with GBI ≥30 % (*p* < 0.001) and VPI ≥20 % (*p* < 0.001) were found to have a significantly stronger risk associated with CP. Similar findings was reported in previous study where having higher GBI and VPI scores were associated with a stronger risk of CP [42]. These findings could be explained by the fact that persistent inflammation and dental plaque accumulation are measures of gingival inflammation [34] and predictors of CP [32]. This is supported by the understanding that dental plaque is an etiological factor for CP and its accumulation is associated with inflammation of the gingival tissues leading to gingivitis, which if left untreated progresses to CP [34]. The current study did not collect information on presence of diagnosed diabetes mellitus and cardiovascular diseases, which are established risk factors for periodontal diseases. The research is not intended to establish obesity as a risk factor for periodontal disease.

There were certain limitations of this study: (i) cross sectional study design, limits our ability to assess association between CP and obesity which can be better done with longitudinal cohort studies, (ii) the small sample size limited our ability for evaluating the relationship of obesity and CP across subgroups (age, gender, ethnicity, educational level, smoking and alcohol intake), (iii) absence of non-obese group, (iv) the body fat percentage was not measured because of financial constraints, logistical problems and patient mobility. The body fat percentage could help us identify the overall distribution of obesity, (v) Furthermore this study had a limited number of smokers which made it difficult to assess the risk of smoking on periodontal health.

The advantages of this study are: (i) using a definite case definition for CP which is universally acceptable, (ii) using FMPM, as it provides the true prevalence and (iii) using the Asian cut off BMI for obesity. This study is reflective of a selected Asian population and could henceforth pave the way for future researchers to relate to our findings.

## Conclusion

This study reported a high prevalence of CP in the obese Malaysian population. In this particular population, the strong predictors for CP found were GBI and VPI.

## Abbreviations

CAL: Clinical attachment loss
CP: Chronic periodontitis
BMI: Body mass index
BOP: Bleeding on probing
FMPM: Full mouth periodontal measurement
GBI: Gingival bleeding index
NOHSA: National oral health survey in adults
PPD: Probing pocket depth
USA: United States of America
VPI: Visible plaque index
WHO: World health organization
